# Impact of Malnutrition on the Outcomes in Patients Admitted with Heart Failure

**DOI:** 10.3390/jcm13144215

**Published:** 2024-07-19

**Authors:** Nahush Bansal, Abdulmajeed Alharbi, Momin Shah, Ibrahim Altorok, Ragheb Assaly, Nezam Altorok

**Affiliations:** 1 Department of Internal Medicine, The University of Toledo, Toledo, OH 43606, USA; 2 College of Art and Science, The University of Toledo, Toledo, OH 43606, USA; 3 Department of Pulmonary and Critical Care Medicine, The University of Toledo, Toledo, OH 43606, USA; 4 Department of Rheumatology, The University of Toledo, Toledo, OH 43606, USA

**Keywords:** malnutrition, heart failure, severe malnutrition, mortality, length of stay, hospital charges, nutritional interventions

## Abstract

**Background:** Heart failure, a major public health concern, significantly contributes to hospital admissions. This study evaluates the impact of malnutrition on both patient and hospital outcomes in heart failure admissions, with a specific focus on variations in outcomes based on the severity of malnutrition. **Methods:** Utilizing the National Inpatient Sample (NIS) database, this retrospective cohort study included adult patients admitted with a principal diagnosis of heart failure. Malnutrition was identified using the well-validated ICD 10 codes. We compared outcomes between patients with and without malnutrition, focusing on mortality, length of stay (LOS), hospital charges, cardiac arrest, and cardiogenic shock. **Results:** Out of 1,110,085 heart failure patients, 36,522 (3.29%) were malnourished. Malnourished patients exhibited significantly higher adjusted in-hospital mortality rates (aOR 3.32; 95% CI 3.03–3.64), longer LOS (mean increase of 4.67 days; *p* < 0.001), and higher hospital charges (mean increase of USD 77,416.9; *p* < 0.01). Increased rates of cardiac arrest (aOR 2.39; 95% CI 1.99–2.86; *p* < 0.001) and cardiogenic shock (aOR 3.74; 95% CI 3.40–4.12; *p* < 0.001) were also noted in malnourished patients. Severely malnourished patients faced worse outcomes compared to those with mild to moderate malnutrition. **Conclusions:** Heart failure patients with malnutrition experience higher mortality rates, longer hospital stays, increased hospitalization charges, and greater complication rates, including cardiac arrest and cardiogenic shock, compared to non-malnourished patients. Outcomes deteriorate with the increasing severity of malnutrition. Timely and individualized nutritional interventions may significantly improve outcomes for heart failure admissions.

## 1. Introduction

Heart failure is a rapidly growing public health concern, with an estimated prevalence of 6.7 million adults in the United States (U.S.) above the age of 20 years [1]. Recent epidemiological data projects a lifetime risk of heart failure at 24%, with approximately 8.5 million cases forecasted by the year 2030 in the U.S. The economic impact is striking, with the expenditure on heart failure care estimated to be USD 43.6 billion in 2020, a burden expected to escalate further with rising prevalence [2].

The World Health Organization (WHO) defines malnutrition as deficiencies or excesses in nutrient intake, an imbalance of essential nutrients, or impaired nutrient utilization. Although by definition, malnourished individuals can be either under- or over-nourished, ‘malnutrition’ is often used synonymously with ‘undernutrition’, as is the case in this article. Malnutrition has been shown to have drastic consequences in various cardiovascular diseases [3,4,5,6,7]. For patients with chronic heart failure, malnutrition holds key prognostic value [8]. However, the evidence is limited in an acute setting. We hypothesized that admitted patients with heart failure and concurrent malnutrition are likely to have significantly worse outcomes compared to patients without malnutrition.

This study evaluates the effect of malnutrition on outcomes, including mortality, length of stay (LOS), and total cost of hospitalization in patients with heart failure using the National Inpatient Sample (NIS) database, which represents all nonfederal acute care hospitals in the U.S. The occurrence of complications, including cardiogenic shock and cardiac arrest, and the differences in outcomes based on the severity of malnutrition, were also assessed.

## 2. Materials and Methods

This is a retrospective cohort study of adult patients hospitalized in 2020 across acute care hospitals in the U.S. The analysis was conducted using the National Inpatient Sample (NIS), created by the Agency for Healthcare Research and Quality (AHRQ). It is the largest publicly available all-payer inpatient database, representing all nonfederal acute care hospitals nationwide. Hospitals are stratified according to ownership, control, bed size, teaching status, urban/rural location, and geographic region. A 20% probability sample of all hospitals within each stratum is then collected. All discharges from these hospitals are recorded and weighted to represent national estimates. The 2020 NIS sampling frame includes data from 49 statewide data organizations, covering 98 percent of the U.S. population, providing both hospital and patient-level information.

Patients were selected using the International Classification of Diseases, Tenth Revision, Clinical Modification (ICD-10-CM) coding system. ICD-10-CM codes for the principal diagnosis of heart failure (I50.xx, I09.81, I11.0, I13.0, I13.2) were identified. All adult patients admitted with heart failure were included in the study. Malnutrition was identified using ICD-10 codes E40-E46, defined as follows:
**ICD-10 Codes****Diagnosis**E40KwashiorkorE41Nutritional marasmusE42Marasmic kwashiorkorE43Unspecified severe protein-calorie malnutritionE44Mild and moderate protein-calorie malnutritionE45Retarded development following protein-calorie malnutritionE46Unspecified protein-calorie malnutrition

These diagnostic codes were used in prior studies [9,10] and were recommended by the Clinical Classifications Software Refined (v2024.1), a software tool that classifies ICD-10-CM codes into clinically meaningful categories from the Healthcare Cost and Utilization Project (HCUP) [11]. E42 and E43 codes were used to represent severe malnutrition, and the remaining codes represent mild and moderate malnutrition. The use of these codes to describe the severity of malnutrition has been defined previously in the literature [10,12]. All the ICD-10-CM codes used in the study for diagnosis and outcomes are listed in Supplementary Table S1. The primary outcome of the study was in-hospital mortality. Secondary outcomes included length of stay (LOS), total hospital charges, occurrence of cardiac arrest, and cardiogenic shock. The exposure of interest was the presence of malnutrition. Outcomes were also compared based on the degree of malnutrition, whether severe or mild to moderate.

Data were analyzed with Software for Statistics and Data Science (STATA/MP 18.0, Stata Corp, College Station, TX, USA). A univariate screen was performed for different outcomes in heart failure patients. Multivariate logistic regression was used to adjust for confounders including age, sex, race, median income, patient comorbidities measured using the Charlson Comorbidity Index (CCI), hospital location (rural or urban), geographic region (Northeast, Midwest, West, or South), hospital academic status, hospital bed size, and primary expected payer or insurance status. Continuous variables were expressed as means (95% CI), and regression analysis was used to compare these differences between malnourished and non-malnourished groups. The chi-squared test was used to compare differences between categorical variables. A two-sided *p* < 0.05 was considered significant throughout the analyses.

## 3. Results

### 3.1. Patient Characteristics

**Figure 1:**
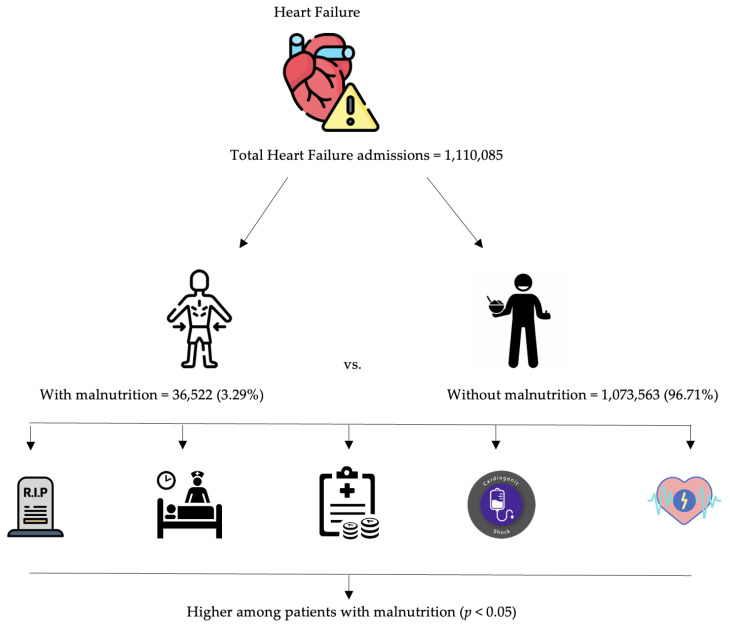
Impact of malnutrition on the in-hospital outcomes of patients admitted with heart failure. Abbreviations: LOS—length of stay.

The NIS 2020 database was used for this study, consisting of 32,355,827 hospitalizations, out of which 1,110,085 patients had a primary diagnosis of heart failure. Of these, 36,522 (3.29%) patients were found to have malnutrition and 1,073,563 (96.71%) patients did not have malnutrition. Severe malnutrition was present in 24,422 (2.20%) patients, while mild to moderate malnutrition was found in 12,100 (1.09%) patients. Compared to heart failure patients without malnutrition, those with malnutrition were more likely to be female, older, and have a higher incidence of chronic conditions, resulting in a higher Charlson Comorbidity Index. Table 1 compares the demographic and hospital-related characteristics of heart failure patients with and without malnutrition. Figure 1 provides an illustration of the outcomes noted in the study.

### 3.2. Primary Outcome: Mortality

**Figure 2:**
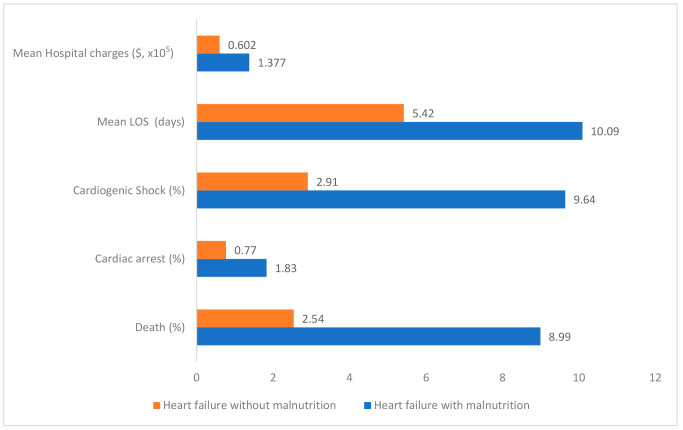
Comparison of outcomes between heart failure patients with and without malnutrition. All results are significant with *p* value < 0.05.

The total in-hospital mortality for patients admitted with heart failure was 2.75% (30,580 patients). Mortality rates for heart failure patients with malnutrition were much higher at 8.99% (9.2% in the severe malnutrition group and 8.61% in the mild to moderate malnutrition group). Both univariable and multivariable analyses, adjusted for patient and hospital-level confounders, showed that heart failure patients with malnutrition had significantly higher odds of mortality (aOR 3.32; 95% CI 3.03–3.64). Figure 2 compares the outcomes between heart failure patients with and without malnutrition. Table 2 compares the outcomes between heart failure patients with mild to moderate and severe malnutrition.

### 3.3. Secondary Outcomes 

#### 3.3.1. Resource Utilization: Length of Stay and Hospital Charges

Resource utilization was analyzed by determining the length of stay and hospital charges. The mean hospital length of stay in heart failure patients with malnutrition was significantly longer, with a mean of 10.09 days (95% CI 9.63–10.55; *p* < 0.001), compared to heart failure patients without malnutrition, who had a mean of 5.42 days (95% CI 5.37–5.48). The mean length of stay was 10.19 days for the severe malnutrition group and 9.86 days for the mild to moderate malnutrition group.

Heart failure patients with malnutrition also had significantly higher hospital charges, with a mean of USD 137,652.7 (95% CI 123,889.1–151,416.4; *p* < 0.01), compared to heart failure patients without malnutrition, who had a mean of USD 60,235.8 (95% CI 58,401.8–62,069.7). These results highlight the significant burden of malnutrition associated with heart failure on healthcare resources. Figure 2 and Table 2 illustrate these findings.

#### 3.3.2. Complications 

**Figure 3:**
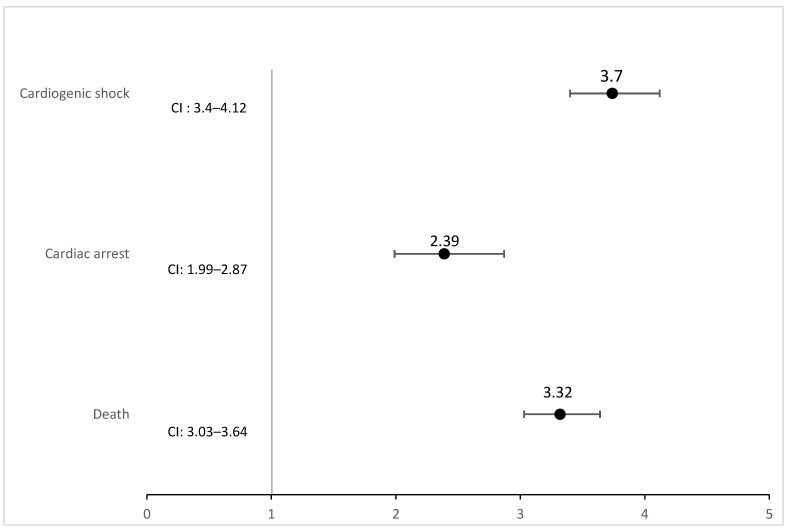
Adjusted odds ratio for the major outcomes in heart failure patients with malnutrition. CI: 95% Confidence Interval.

After adjusting for patient and hospital-level confounders, heart failure patients with malnutrition had significantly higher rates of cardiac arrest (aOR 2.39; 95% CI 1.99–2.86; *p* < 0.001) compared to patients without malnutrition. Furthermore, patients with malnutrition also had significantly higher rates of cardiogenic shock (aOR 3.74; 95% CI 3.40–4.12; *p* < 0.001) compared to heart failure patients without malnutrition (Figure 3).

## 4. Discussion

This population-based nationwide study analyzed the impact of malnutrition on in-hospital outcomes in patients admitted with heart failure to U.S. hospitals. To our knowledge, this is the first study of its kind using the national inpatient database in the United States. The main findings of this study were that malnutrition in heart failure patients showed a prevalence of 3.29%, of which 2.20% had severe malnutrition. Heart failure patients with malnutrition were found to have higher rates of in-hospital mortality, higher risk of complications including cardiogenic shock and cardiac arrest, as well as longer lengths of stay (LOS) and significantly higher hospitalization costs. Interestingly, our data also demonstrated that the impact of malnutrition on hospital and patient outcomes was proportionate to the degree of malnutrition. Specifically, severe malnutrition was associated with worse outcome measures compared to patients with mild to moderate malnutrition.

In our study, malnutrition was associated with approximately three times the increased risk of in-hospital mortality for patients admitted with heart failure. Malnutrition has previously been linked to a significant increase in mortality across various cardiovascular conditions, including acute myocardial infarction, cardiomyopathy, and atrial fibrillation [3,4,5,6,7]. In a large-scale French multicenter study involving 619,805 chronic heart failure patients, malnutrition was associated with a significant decrease in life expectancy at the one-year (aHR = 1.16) and four-year follow-ups (aHR = 1.04) [13]. Scotti et al. evaluated patients with heart failure associated with mitral regurgitation and demonstrated that baseline malnutrition was an independent predictor of mortality [14]. Similarly, a prospective study involving 9733 chronic heart failure patients showed that malnutrition was strongly associated with mortality, independent of the diagnostic tool used for malnutrition assessment and the type of heart failure [15]. Moderate to severe malnutrition has been identified as a key risk factor for postoperative mortality in heart failure patients undergoing left ventricular assist device (LVAD) implantation [16].

Along with mortality, malnutrition was associated with increased cardiac complications and resource utilization, measured by in-hospital length of stay and hospitalization charges. Several mechanisms can potentially contribute to these adverse outcomes. The phenomenon known as “the obesity paradox,” where less body fat has detrimental effects on the prognosis of heart failure patients, has been illustrated in several heart failure studies previously [17,18]. High levels of lipids and cholesterol in the circulatory system can bind to endotoxins, resulting in the inhibition of their harmful effects. Furthermore, since heart failure is a well-known catabolic state, having fewer metabolic reserves explains the less favorable prognosis in malnourished patients. Low lean mass drastically affects hemostasis and is linked to myocyte metabolic dysregulation, potentially causing the progression of heart failure [19]. The presence of malnutrition in heart failure signifies high activation of neurohormonal and inflammatory pathways, forming the basis of cardiac cachexia. Increased expression of inflammatory cytokines, including IL-1, IL-6, IL-18, TNF-α, and cardiotrophin-1 (CT-1), has been shown to directly impair cardiac function and stimulate hypertrophy and fibrosis, leading to cardiac remodeling [20]. Recent studies have even associated hypoalbuminemia, an important marker of malnutrition, with systolic and diastolic myocardial dysfunction by causing endothelial dysfunction and triggering inflammation [21].

Given the significant impact of malnutrition on heart failure patients, it is crucial to make early and adequate assessments of the nutritional status of these patients. Various tools have been developed for this purpose, including questionnaires, laboratory parameters, scoring systems, and body composition assessment techniques. The Nutritional Risk Screening 2002 (NRS 2002) is a simple four-point questionnaire that provides quick and effective screening of nutritional status based on recent weight loss, body mass index (BMI), and dietary intake, along with the severity of the underlying disease. A study of 588 patients with advanced heart failure undergoing cardiac implant electronic devices showed that NRS 2002 is a significant predictor of mortality and length of stay in patients requiring CRT-D implants [22]. Assessment of body composition plays a crucial role in determining the overall health of patients with cardiovascular conditions [23]. Several basic and advanced techniques have been used to adequately measure body fat and lean mass, each providing different levels of accuracy. Skinfold thickness measurements using calipers at various sites (e.g., triceps, abdomen) help in determining total body fat percentage. Bioelectrical Impedance Analysis (BIA) is another non-invasive technique that measures the resistance and reactance of electrical currents as they pass through body tissues, providing estimates of body fat, muscle mass, and overall hydration status. Dual-energy X-ray Absorptiometry (DEXA) uses low-level X-rays to accurately differentiate between bone, fat, and muscle tissue. Advanced imaging techniques like CT scans and MRI can provide precise differentiation of body tissues but are expensive and involve radiation and contrast exposure. Hydrostatic weighing and Air Displacement Plethysmography (ADP) are traditional methods of estimating composition through tissue density measurements by placing the patient under water and in a chamber, respectively.

Various tools are also used to determine the prognosis in malnourished patients with heart failure. A recent meta-analysis demonstrated that the Mini Nutritional Assessment Short Form (MNA-SF) is the most robust predictor of overall mortality in heart failure patients, based on both quantitative and qualitative aspects [24]. Some other relevant tools include the Controlling Nutritional Status (CONUT), which sums the albumin, cholesterol, and lymphocyte count; the Prognostic Nutritional Index (PNI), calculated as albumin (g/L) + 5 × total lymphocyte count (×10^9^/L); and the Geriatric Nutritional Risk Index (GNRI), defined as (1.489 × albumin (g/L)) + 41.7 × (actual weight/ideal weight). All have shown a positive association with mortality. Addressing malnutrition early with an individualized approach and supplements may play a key role in improving patient outcomes, although more studies are needed to establish firm recommendations.

There are several limitations to our study. Firstly, as a retrospective study relying on ICD-10 codes, it is subject to misclassification bias. However, the ICD-10 coding for malnutrition has demonstrated high accuracy and positive predictive value [25,26]. This study utilized an administrative database, which does not allow for accurate determination of illness severity. This issue was addressed by incorporating the Charlson Comorbidity Index, a commonly used and validated prognostic scale [27]. Finally, certain important parameters such as symptoms, laboratory values, the etiology of heart failure, causes of mortality, and treatments administered cannot be analyzed using the NIS. Despite these limitations, the substantial amount of administrative data in the NIS provides adequate power to draw safe conclusions between the groups.

## 5. Conclusions

Malnutrition significantly affects in-hospital outcomes in patients admitted with heart failure, with approximately a threefold increased risk of mortality and heightened risk of severe complications, including cardiogenic shock and cardiac arrest. Heart failure patients with malnutrition also place a substantial burden on healthcare resources, evidenced by increased lengths of stay and hospitalization charges. The impact on these outcomes is proportional to the degree of malnutrition, with severe malnutrition associated with worse outcomes compared to mild to moderate malnutrition. Early diagnosis and interventions for malnutrition can potentially play a crucial role in improving outcomes for patients admitted with heart failure. Given these findings, further research is necessary to analyze the impact of individualized nutritional support on improving both short and long-term outcomes in patients with heart failure.

## Figures and Tables

**Table 1:** Demographics and hospital characteristics of patients with acute heart failure with and without contaminant malnutrition.

Variable	With Malnutrition	Without Malnutrition	*p* Value *
% of Patients with Heart Failure	3.29	96.71	
Mean Age (years)	74.43	70.36	<0.001
Female Gender (%)	48.68	46.47	<0.001
**Race (%)**			<0.001
Caucasian	67.1	64.5	
African American	19.5	22.1	
Hispanic	7.4	8.3	
Asian or Pacific Islander	2.8	2.1	
Native American	0.7	0.6	
Others	2.5	2.3	
**Median Income in Patient’s Zip Code (%)**			<0.001
USD 1–USD 47,999	31.1	34.5	
USD 48,000–USD 60,999	26.2	27.9	
USD 61,000–USD 81,999	22.4	21.4	
≥USD 82,000	20.4	16.3	
**Charlson Comorbidity Index (%)**			<0.001
0	0	0	
1	6.6	8.6	
2	13.1	14.4	
3 or more	80.3	77	
**Hospital Region**			<0.001
Northeast	19.5	17.7	
Midwest	23.9	22.8	
South	36.4	42.1	
West	20.2	17.4	
**Hospital Bed Size (%)**			
Small	20.8	24.3	
Medium	27.1	28.9	
Large	52.2	46.9	
**Hospital Location (%)**			0.31
Rural	7.4	10.4	
Urban	92.6	89.6	
**Hospital Teaching Status (%)**			
Non-Teaching	25.8	29.4	
Teaching	74.2	70.6	
**Comorbidities (%)**			<0.001
Diabetes	7.5	13.5	
Diabetes with Complications	29.4	37.3	
COPD	39.4	40.5	
Dementia	13.9	7.1	
Cancer	7	3.6	
Renal Dysfunction	57.5	54.9	
Peptic Ulcer Disease	1.6	0.8	

* *p* value ≤ 0.05 indicates significance.

**Table 2:** Comparison of outcomes in heart failure patients with mild to moderate malnutrition and heart failure patients with severe malnutrition.

Variables/Outcomes	Heart Failure with Mild to Moderate Malnutrition	Heart Failure with Severe Malnutrition	*p* Value *
n (% of total heart failure patients)	12,211 (1.1)	24,422 (2.2)	
Death (%)	8.61	9.20	0.05
Cardiac arrest (%)	1.61	2.25	<0.01
Cardiogenic shock (%)	9.38	9.75	0.25
Mean LOS in days (95% CI)	9.86 (9.14–10.59)	10.19 (9.64–10.74)	0.49
Mean total charges USD (95% CI)	136,782.5 (117,635.8–155,929.3)	137,923.4 (121,145–154,701.9)	0.93

* *p* value ≤ 0.05 indicates significance.

## Data Availability

Data are available upon request through the corresponding author.

## Supplementary Materials

The following supporting information can be downloaded at: https://www.mdpi.com/article/10.3390/jcm13144215/s1, Table S1: ICD 10-CM codes used for various conditions in the study.
