# Supplementary material for: Impact of Malnutrition on the Outcomes in Patients Admitted with Heart Failure
Source: J Clin Med. 2024 Jul 19;13(14):4215. doi: 10.3390/jcm13144215 (PMC11278307; doi:10.3390/jcm13144215)
Supplement: Supplementary file 1 [file jcm-13-04215-s001.zip › jcm-3084666-supplementary.pdf]

Supplementary Table S1: ICD 10-CM codes used for various conditions in the study.

**Condition**	**ICD10-CM codes**
Heart Failure	I50xx, I0981, I110, I130, I132
Malnutrition	E40, E41, E42, E43, E44, E45, E46
Severe Malnutrition	E42, E43
Mild to Moderate Malnutrition	E40, E44, E45, E46
Cardiac arrest	I462, I468, I469
Cardiogenic shock	R570
